# Associations of body composition and resting metabolic rate with homeostatic and hedonic components of appetite

**DOI:** 10.1038/s41366-026-02115-1

**Published:** 2026-06-02

**Authors:** Arwa Alruwaili, Hannah Trotman, Laure Haenebalcke, Adrian Sia Jin Chuen, Melanie Partington, Bethan Roberts, Buket Engin, Sundus Malaikah, Mark Hopkins, Graham Finlayson, James Sanders, Alice E. Thackray, David J. Stensel, Scott A. Willis, James A. King

**Affiliations:** 1 National Centre for Sport and Exercise Medicine, School of Sport, Exercise and Health Sciences, Loughborough University, Loughborough, UK; 2 NIHR Leicester Biomedical Research Centre, University Hospitals of Leicester NHS Trust and the University of Leicester, Loughborough, UK; 3 Department of Respiratory Therapy, College of Applied Medical Sciences, King Saud bin Abdulaziz University for Health Sciences, Riyadh, Saudi Arabia; 4 King Abdullah International Medical Research Center, Riyadh, Saudi Arabia; 5 Department of Physiotherapy and Rehabilitation, Faculty of Health Sciences, Afyonkarahisar Health Sciences University, Afyonkarahisar, Turkey; 6 Clinical Nutrition Department, Faculty of Applied Medical Sciences, King Abdulaziz University, Jeddah, Saudi Arabia; 7 School of Food Science & Nutrition, Faculty of Environment, University of Leeds, Leeds, UK; 8 School of Psychology, Faculty of Medicine and Health, University of Leeds, Leeds, UK; 9 Centre for Lifestyle Medicine and Behaviour, Loughborough University, Loughborough, UK; 10 Faculty of Sport Sciences, Waseda University, Waseda, Japan; 11 Department of Sports Science and Physical Education, The Chinese University of Hong Kong, Shui, Hong Kong

**Keywords:** Obesity, Risk factors

## Abstract

**Background:**

While fat-free mass (FFM), fat mass (FM), and resting metabolic rate (RMR) influence energy intake, their specific roles as homoeostatic and hedonic drivers of eating—and factors moderating these relationships—are uncertain. This study examined associations of FFM, FM, and RMR with a comprehensive set of appetite-related outcomes in adults.

**Methods:**

130 participants (58% male; age: 25.0 ± 8.5 years; BMI: 24.0 ± 3.7 kg/m²; mean ± SD) completed assessments of RMR (indirect-calorimetry), body composition (air-displacement plethysmography), energy intake (laboratory-based), appetite-related hormones (fasted and meal-stimulated), appetite (visual analogue scales), food reward (Leeds Food Preference Questionnaire [LFPQ]), food cravings (Control of Eating Questionnaire), taste and smell perception. GLM examined associations between exposure and outcome variables, controlling for demographic and lifestyle factors. Effect modification was assessed via interaction terms and stratified analyses with median splits for continuous variables.

**Results:**

RMR was positively associated with energy intake (*P* < 0.001), while FFM and FM were not. The positive RMR-energy intake association was stronger in individuals who were younger, more active, smokers, and with lower BMI (*P* ≤ 0.020). FFM was positively associated with postprandial peptide-YY (PYY) and hunger, craving control and smell sensitivity (*P* ≤ 0.026). FM was positively associated with fasting PYY and leptin, and inversely associated with postprandial PYY and hunger, craving control, and taste sensitivity (*P* ≤ 0.047). RMR was inversely associated with fasting ghrelin (*P* < 0.001). No consistent associations were observed between body composition, RMR and food reward (LFPQ). Several associations were moderated by age, sex, BMI, physical activity and smoking.

**Conclusions:**

RMR is a key driver of energy intake, with sex, BMI, physical activity and smoking status moderating the relationship. Body composition (FFM and FM) is linked to distinct variations in homoeostatic and hedonic components of appetite, particularly gut hormone responses, craving control and taste perception.

## Introduction

Recent research has identified fat-free mass (FFM) as a key physiological driver of energy intake, complementing traditional models of appetite regulation emphasising inhibitory signals from fat mass (FM) and the gastrointestinal tract [1]. While FM may exert a tonic inhibitory influence on energy intake (at least in healthy-weight individuals) [2], FFM appears to stimulate energy consumption; an effect seemingly linked to energy requirements [3, 4]. This drive appears to act largely via resting metabolic rate (RMR) and/or total daily energy expenditure (TDEE) [5]. Studies show that associations of FFM, FM and RMR with energy intake are modified (stronger or weaker) in certain populations, adding further complexity to appetite control and weight management [6]. For example, older adults exhibit a weaker (positive) relationship between FFM and energy intake, potentially contributing to the ‘*anorexia of ageing*’ [7]. Whether broader demographic, anthropometric and lifestyle factors moderate associations requires clarification.

Associations of FFM and RMR with daily energy intake have been well established (in weight-stable individuals), while the findings for FM are conflicting. However, progress in understanding these relationships has been hindered by challenges in identifying underlying mechanisms. While complex biochemical, neural, and metabolic pathways have been proposed [8], evidence is lacking. Moreover, it is unclear whether FFM, FM, and RMR are linked to more specific aspects of eating behaviour. This is crucial as daily energy intake reflects eating behaviour, i.e. decisions about when, what, and how much to eat, which is heavily influenced by appetite, including homoeostatic and hedonic components. Despite these links, no studies have systematically examined whether FFM, FM, and RMR independently relate to appetite perceptions, appetite-related peptides, or hedonic drivers of eating.

Contemporary models of appetite control emphasise neuro-humoral components involving circulating peptides [9]. Acylated ghrelin (appetite-stimulating) [10] and peptide-YY (PYY, appetite-suppressing) [11] are prominent gut peptides which help coordinate appetite, food intake, and digestion on a meal-to-meal basis. Synergistically, the adipose-derived peptide leptin helps regulate long-term energy balance [12]. Its appetite-suppressing effects may contribute to the inverse association between FM and energy intake (reported in some studies) [3], and leptin resistance (central) may explain the weakening of this association with obesity [3]. Further research is needed to clarify how FFM, FM, and RMR relate to these peptides and to identify potential moderators of these associations.

Hedonic factors also influence food intake based on pleasure, taste, enjoyment, and psychological reward [13], and may override metabolic stimuli. Several studies have found that obesity is associated with altered food preferences [14], cravings [15], smell [16] and taste sensitivity [17], potentially suggesting a relationship between FM and these outcomes. However, since FFM and RMR also increase with obesity, disentangling their independent associations with hedonic drivers warrants further investigation.

To advance understanding, we conducted a large cross-sectional study with in-depth phenotyping of homoeostatic and hedonic drivers of appetite and eating behaviour. We aimed to: (1) document associations of FFM, FM and RMR with energy intake; and specifically determine whether demographic, anthropometric and lifestyle factors moderate these associations; (2) examine associations of FFM, FM and RMR with appetite perceptions, appetite-related hormones, and hedonic eating influences (food preferences, cravings, taste and smell sensitivity).

## Methods

### Ethical approval and participants

This study was approved by the local Ethics Review Sub-Committee (project ID: 10980; 30/09/2022) and all methods were performed in accordance with the relevant guidelines and regulations. 130 participants (76 males, 54 females) aged 18–55 years with a BMI of 16.5–35.0 kg/m² were recruited from university students, staff, and the local community. All were healthy, weight-stable (<3 kg change in 12-weeks), non-dieting, not using e-cigarettes or weight-affecting medications, and had no metabolic or cardiovascular conditions. Females self-reported not being pregnant. Written informed consent was obtained and procedures followed the Declaration of Helsinki [18].

### Study design

This cross-sectional study involved two laboratory visits, separated by 1-week during which free-living physical activity and sedentary time were monitored. Participants fasted overnight (12 h) and abstained from caffeine, alcohol, and exercise for 24-h before visits.

### Visit 1: Anthropometry, RMR and cardiorespiratory fitness (CRF)

Participants attended the laboratory to confirm eligibility and for familiarisation with study procedures. They also completed assessments for demographic outcomes, anthropometry, RMR, taste and smell sensitivity. Food cravings over the previous week were examined using the Control of Eating Questionnaire (CoEQ) [19]. Taste and smell perceptions were assessed using commercially available taste strips and Sniffin’ Sticks (Burghart Messtechnik, Germany) with established reliability and validity [20–22] (see Supplementary Methods for additional detail).

Height and body mass were measured to the nearest 0.1 cm and 0.1 kg, respectively, using a wireless measuring station (Seca Ltd, Hamburg, Germany), with subsequent calculation of body mass index (BMI). FFM and FM were assessed using air displacement plethysmography (BOD POD, Life Measurement, Inc., Concord, USA) according to manufacturer guidelines, with participants wearing minimal clothing (e.g. tight-fitting swimwear) and a tight-fitting nylon cap.

RMR was measured following published guidance [23] using breath-by-breath indirect calorimetry (Cortex Metalyzer 3B, Leipzig, Germany). Participants lay still in a calm, awake, and quiet supine position for 30-min. Expired air was continuously sampled to measure oxygen uptake, carbon dioxide production, and energy expenditure. The first 10-min were excluded to allow for steady-state conditions, and RMR (kcal·day^−1^) was calculated from the remaining 20-min using the Weir equation [24]. CRF (*V̇*O_2_ peak) was also determined via indirect calorimetry during an incremental treadmill test to voluntary exhaustion.

### Free-living physical activity and sedentary time assessment

After Visit 1, free-living physical activity and sedentary time were monitored for seven consecutive days using a triaxial accelerometer (GENEActiv, ActivInsights Ltd, UK) worn on the non-dominant wrist. Participants maintained their usual activity patterns and recorded sleep/wake times and any device removal. Data were recorded at 100 Hz, downloaded via GENEActiv software (v3.3), and processed using the GGIR R-package (v3.1.2) [25]. Dynamic acceleration, adjusted for gravity (Euclidean Norm minus 1 *×* *g*), was averaged over five-second epochs and expressed in milligravitational units (m*g*). Days with <16 h wear time were excluded; ≥4 valid days were required for inclusion. Movement behaviours were calculated as average minutes/day and categorised as sedentary time (<40-m*g*, excluding sleep), light intensity physical activity (LPA; 40–<100-m*g*) or moderate-vigorous intensity physical activity (MVPA; ≥100-m*g*) [26].

### Visit 2: Appetite and energy intake assessment

**Fig. 1:**
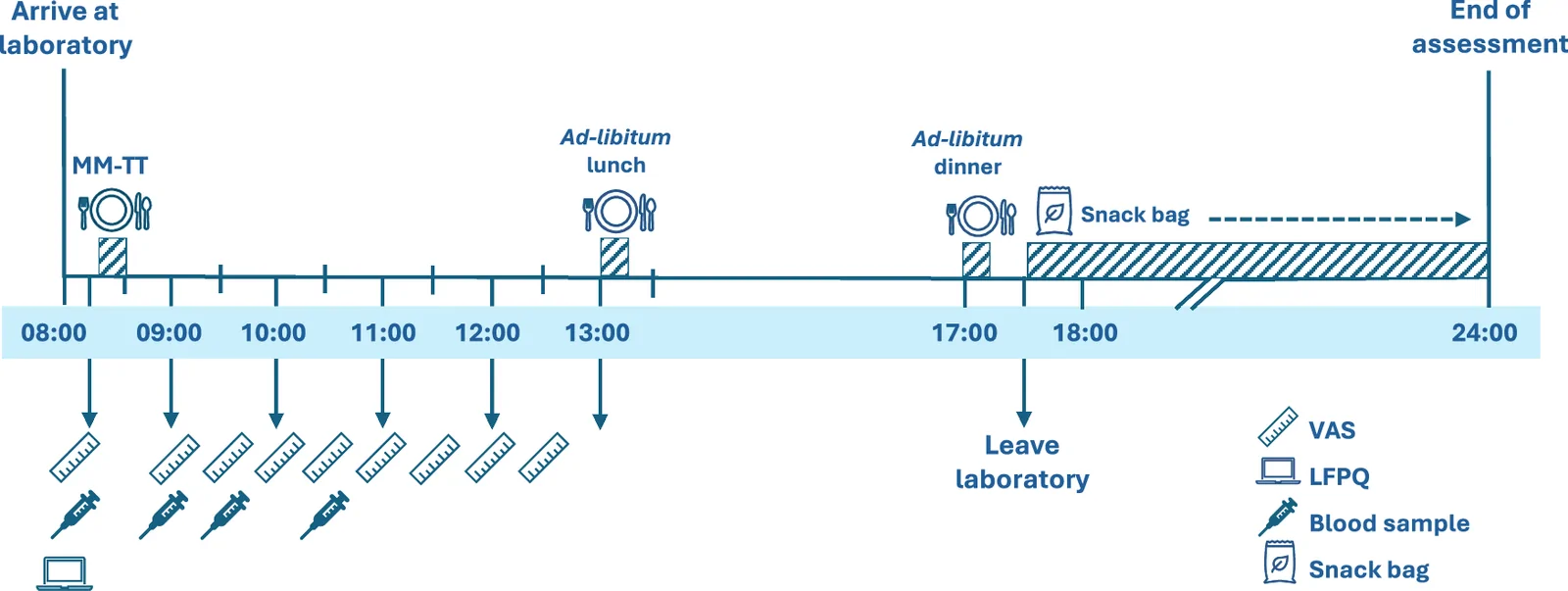
Schematic overview of Visit 2. LFPQ, Leeds Food Preference Questionnaire; MM-TT mixed-meal tolerance test, VAS visual analogue scale. Reproduced from Alruwaili et. al., *Appetite* 2025; Jun 11:108194, under the terms of the Creative Commons Attribution (CC BY 4.0) license. https://doi.org/10.1016/j.appet.2025.108194.

For Visit 2, participants arrived at the laboratory at 08:00 after 12 h of fasting. A schematic overview of the study visit is presented in Fig. 1 [27]. On arrival, perceived ratings of appetite (hunger, fullness, satisfaction, and prospective food consumption [PFC]) were assessed using 100-mm pen and paper visual analogue scales (VAS) [28], while the Leeds Food Preference Questionnaire (LFPQ) [29] was completed to assess food preference and reward. A fasting venous blood sample was then collected via venepuncture of an antecubital vein. Participants then undertook a 4-h mixed-meal tolerance test (MM-TT), with venous blood samples collected at 30-, 60- and 120 min post-meal, while VAS were completed at 30-min intervals over 240 min.

Participants consumed *ad libitum* lunch (13:00) and dinner (17:00) meals before leaving the laboratory (~17:30) with a snack bag, from which they could eat until the next morning. Food intake from the snack bag (up to midnight) was recorded via follow-up the next day.

### Study meals

The MM-TT meal consisted of porridge, whole milk, and honey (55% carbohydrates, 15% protein, 30% fat). Meal energy was determined by multiplying participants' RMR by 1.4 (to reflect the sedentary study conditions) and by 0.25 (typical share of daily energy intake from breakfast in the UK) [30]. Participants consumed the meal within 15-min.

Laboratory measured energy intake included test meal energy intake plus *ad libitum* energy intake from lunch, dinner meals and the evening snack bag. The *ad libitum* lunch consisted of a homogeneous meal of pasta, tomato sauce, and olive oil (72% carbohydrate, 12% protein, 16% fat; 6.5 kJ/g) [31]. Participants self-served from a Pyrex^®^ bowl containing roughly 1 kg of cooked pasta and ate in isolation until comfortably full. Energy intake was calculated by subtracting the weight of any leftovers from the initial serving. Manufacturer values were used to determine macronutrient and energy intake. The *ad libitum* dinner comprised curry sauce, plain naan, and microwaveable basmati rice (71% carbohydrates, 8% protein, 21% fat). Serving and energy intake measurements were performed as for lunch. The evening (outside of lab) snack bag contained Maryland cookies, Kellogg’s Nutri-Grain^®^ bars, and Cadbury Chocolate Mini-rolls^®^.

### Leeds food preference questionnaire

The LFPQ is a laptop-based tool for measuring food preference and reward [29, 32]. It assesses liking and wanting for 16-food items across four categories: high-fat savoury, low-fat savoury, high-fat sweet, and low-fat sweet. Relative preference is determined by selecting the food ‘most wanted‘ from paired items, with reaction times (adjusted for selection frequency) providing an implicit wanting measure. Explicit liking and wanting are rated using a 100-mm VAS, and bias scores for fat and sweet appeal are calculated by subtracting low-fat from high-fat scores and savoury from sweet scores, respectively.

### Blood sampling and biochemical analyses

Venous blood samples were collected into pre-chilled EDTA monovettes (Sarstedt, Leicester, UK) and immediately centrifuged at 2383 *×* *g* for 10-min (4 °C). Plasma was aliquoted into 2 mL cryovials and stored at −80 °C. For acylated ghrelin, separate samples underwent additional steps to preserve the acyl moiety [33]. Plasma acylated ghrelin, total PYY, and leptin concentration were measured using commercially-available enzyme-linked immunosorbent assays (Bioquote Ltd, York, UK; Merck Millipore, Darmstadt, Germany; and Bio-Techne Ltd, Abingdon, UK, respectively) with within-batch CVs of 5.14%, 4.60%, and 3.93%. Acylated ghrelin and total PYY were assessed fasted and postprandially (0, 30, 60, and 120 min) while leptin was measured only at baseline (fasted).

### Statistical analyses

Data were analysed in SPSS v28 (SPSS Inc., Chicago, IL). Distributions were assessed via Kolmogorov–Smirnov tests and histograms. Results are reported as mean ± SD (normal), median (IQR) (non-normal), or *n* (%) (categorical). Total AUC for postprandial appetite and hormone responses (MM–TT) was calculated using the trapezoid rule.

Generalised-linear-models (GLMs) assessed associations of FFM, FM and RMR with appetite-related variables. GLMs employed a normal distribution with identity link function for most variables, while a gamma distribution with log-link function (for fasted ghrelin and leptin) or identity-link function (for taste scores) was used for skewed data, depending on the best model fit (based on the Akaike Information Criterion). Models were adjusted for covariates as follows—Model 1: demographic variables (age, sex, ethnicity), Model 2: previous adjustments + lifestyle variables (CRF, MVPA, accelerometer wear time, and smoking status (defined as an individual having smoked ≥100 cigarettes in their lifetime and regularly smoking ≥5 cigarettes each day)). Postprandial AUCs were further adjusted for fasting values.

Missing data (3.1% fasting blood; 1.5% appetite, activity, energy intake) were imputed across 20 datasets using regression models with age, sex, ethnicity, BMI, and smoking as predictors. Moderation was assessed via interaction terms (age, sex, BMI, MVPA, smoking), with each individual term being added to Model 2 in separate models. To aid interpretation of significant interactions, stratified analyses were also conducted for categorical moderators (sex, smoking), while median splits were applied to continuous moderators (age, BMI, MVPA). All other variables were entered into the models as specified previously. Due to the exploratory focus of the study, power calculations were not performed, and adjustments for multiple testing were not made. As such, findings should be interpreted with caution (due to the potential for Type I error) and considered in relation to broader trends using beta-coefficients (*β*), 95% confidence intervals (CIs), and exact *P*-values.

## Results

### Participant characteristics

Participants characteristics (*n* = 130) are presented in Table 1. Participants were generally young, active and fit (aerobically), and of healthy weight. Furthermore, they were predominantly non-smokers and of white European ethnicity, with a relatively even balance of males and females.

**Table 1:** Participant characteristics.

Characteristic	All (*n* = 130)
Age (years)	25.0 (8.5)
Sex	
Male	76 [58.5]
Female	54 [41.5]
Ethnicity	
White	59 [45.4]
Asian	27 [20.8]
Indian	31 [23.8]
Mixed	6 [4.6]
Black	3 [2.3]
Arab	2 [1.5]
Latino	2 [1.5]
Body mass (kg)	70.5 ± 12.6
BMI (kg·m^−2^)	24.0 ± 3.7
Fat mass (kg)	17.2 ± 9.2
Fat-free mass (kg)	76.0 ± 10.9
RMR (kcal∙day^−1^)	1764 (442)
*V̇*O_2_ peak (mL·kg·min⁻¹)	44.5 ± 9.5
Physical activity	
Daily steps	11,012 ± 3248
MVPA (min·day⁻¹)^a^	24 ± 11
Smoking status	
Non-smoker	98 [75.4]
Smoker	32 [24.6]
Energy intake (*ad libitum*, kcal)	
Total day	2461 ± 700
Lunch	865 ± 346
Dinner	803 ± 288
Snack bag	293 ± 211

Data are presented as mean ± SD for normally distributed continuous data or median (interquartile range) for non-normally distributed continuous data, while categorical data are presented as number [column percentage].

*BMI* body mass index, *LPA* light physical activity, *MVPA* moderate to vigorous physical activity, *RMR* resting metabolic rate, *V̇*O_2_ *peak* peak oxygen uptake.

^a^MVPA data are 5 min bouted to eliminate inclusion of incidental activity.

Table 2 shows the associations of RMR, FFM, and FM with energy intake, circulating appetite-related hormones, perceived ratings of appetite, taste and smell scores, and control of eating (CoEQ) for the finally adjusted model (Model 2). All results for Model 1 are presented in Supplementary Table 1. Data for taste and smell scores were only available in a sub-set of participants (*n* = 62).

**Table 2:** Associations of resting metabolic rate, fat-free mass and fat mass with energy intake, appetite-related hormones, perceived ratings of appetite, taste and smell, and control of eating, in the finally adjusted models (Model 2).

	Resting metabolic rate (kcal day^−1^)		Fat−free mass (kg)		Fat mass (kg)	
	β (95% CI)	*P−*value	β (95% CI)	*P−*value	β (95% CI)	*P−*value
Energy intake						
Lab energy intake (kcal day^−1^)	5.7 (2.4, 9.1)	<0.001	3.1 (−7.9, 14.2)	0.578	1.5 (−10.5, 13.4)	0.810
Appetite−related hormones						
Fasting ghrelin (pg mL^−1^)^b^	−1.00 (−1.54, −0.45)	<0.001	0.00 (−0.02, 0.01)	0.477	0.00 (−0.02, 0.02)	0.935
Ghrelin AUC (2 h, pg mL^−1^)	1.77 (−28.30, 31.85)	0.908	0.00 (−0.01, 0.01)	0.928	19.24 (−121.94, 83.46)	0.713
Fasting PYY (pg mL^−1^)	0.00 (0.00, 0.00)	0.828	−0.79 (−1.83, 0.25)	0.134	1.13 (0.02, 2.24)	0.047
PYY AUC (2 h, pg mL^−1^)	0.00 (−0.02, 0.00)	0.857	136.96 (55.71, 218.20)	<0.001	−139.89 (−228.71, −51.04)	0.002
Fasting leptin (pg mL^−1^)^b^	0.00 (0.00, 0.01)	0.147	−0.04 (−0.05, −0.03)	<0.001	0.06 (0.05, 0.08)	<0.001
Perceived ratings of appetite						
Fasting fullness (mm)	0.03 (−0.10, 0.17)	0.653	−0.31 (−0.74, 0.11)	0.148	0.39 (−0.07, 0.85)	0.097
Fullness AUC (4 h, mm)	3.19 (−22.13, 28.51)	0.805	−44.44 (−124.88, 36.00)	0.279	42.55 (−44.71, 129.81)	0.339
Fasting hunger (mm)	−0.06 (−0.22, 0.10)	0.439	−0.08 (−0.59, 0.43)	0.759	0.10 (−0.45, 0.65)	0.724
Hunger AUC (4 h, mm)	6.67 (−20.90, 34.25)	0.635	97.08 (11.46, 182.71)	0.026	−96.85 (−189.69, −4.01)	0.041
Fasting PFC (mm)	−0.15 (−0.25, −0.04)	0.006	0.13 (−0.22, 0.47)	0.470	−0.14 (−0.51, 0.24)	0.475
PFC AUC (4 h, mm)	7.64 (−16.39, 31.68)	0.533	68.94 (−4.33, 142.21)	0.065	−63.76 (−143.25, 15.72)	0.116
Fasting satisfaction (mm)	0.03 (−0.12, 0.18)	0.705	0.25 (−0.21, 0.78)	0.283	−0.29 (−0.79, 0.21)	0.254
Satisfaction AUC (4 h, mm)	18.23 (−6.60, 43.07)	0.150	−29.53 (−108.97, 49.91)	0.466	29.58 (−56.37, 115.54)	0.500
Control of eating						
Craving control	−0.03 (−0.07, 0.02)	0.205	1.52 (0.22, 2.82)	0.022	−1.61 (−3.02, 0.20)	0.025
Craving for sweet	0.00 (−0.05, 0.05)	0.922	0.85 (−0.78, 2.49)	0.305	−0.79 (−2.55, 0.98)	0.383
Craving for savoury	0.02 (−0.03, 0.07)	0.406	−0.58 (−2.09, 0.93)	0.452	0.78 (−0.85, 2.41)	0.350
Positive mood	0.02 (−0.03, 0.06)	0.459	1.05 (−0.22, 2.31)	0.105	−0.91 (−2.29, 0.46)	0.192
Taste and smell (*n* = 62)						
Taste score^a^	0.00 (−0.05, 0.02)	0.355	0.01 (−0.01, 0.02)	0.313	−0.20 (−0.29, −0.10)	<0.001
Smell score	0.00 (−0.01, 0.02)	0.633	0.07 (0.01, 0.12)	0.026	−0.07 (−0.14, 0.00)	0.058

Data were analysed using generalised linear models and are presented as beta-coefficients (*β*) with 95% confidence intervals (CI). Models for most outcomes used a normal distribution and identity link function, however, based on model fit, skewed outcomes used a gamma distribution with either an identity link function^a^ or log link function^b^. Model 2 was adjusted for age, sex, ethnicity, fasting values (for AUC variables), CRF, MVPA, accelerometer wear time, and smoking status.

*AU* arbitrary units, *AUC* area under the curve, *PFC* prospective food consumption, *PYY* peptide YY, ^b^*β*coefficients for fasting leptin and fasting ghrelin are shown as log-transformed value.

### Energy intake

In Model 2 (Table 2), RMR was positively associated with lab energy intake (5.7 (2.4, 9.1) kcal day^−1^, *P* < 0.001). FFM and FM were not associated with lab energy intake (*P* ≥ 0.578). These results were similar in Model 1 (Supplementary Table 1).

### Appetite-related hormones

RMR was inversely associated with fasting ghrelin concentrations (−1.00 (−1.54, 0.45), *P* < 0.001)(Table 2). FFM was positively associated with PYY AUC (137.0 (55.7, 218.2) 2 h, pg mL^−1^, *P* < 0.001) and inversely related to circulating leptin concentrations (−0.04 (−0.05, −0.03), *P* < 0.001). In contrast, FM showed positive associations with both circulating leptin (0.06 (0.05, 0.08), *P* < 0.001) and fasting PYY concentrations (1.13 (0.02, 2.24) pg mL^−1^, *P* = 0.047) but was inversely associated with PYY AUC (−139.9 (−228.7, −51.0) 2 h, pg mL^−1^, *P* = 0.002).

### Perceived ratings of appetite

In Model 2 (Table 2), RMR was inversely associated with fasting PFC (−0.15 (−0.25, −0.04) mm, *P* = 0.006) (Table 2). FFM was positively associated with hunger AUC (97.08 (11.46, 182.71) 4 h, mm, *P* = 0.026) while FM was inversely related to hunger AUC (−96.80 (−189.7, −4.0) 4 hmm, *P* = 0.041). There were no other associations of RMR, FFM, and FM with fasting or postprandial perceived ratings of appetite. These results were similar in Model 1 (Supplementary Table 1).

### Control of eating (CoEQ)

In Model 2, FFM was positively related to craving control (1.52 (0.22, 2.82) AU, *P* = 0.022), while FM was inversely related to craving control (−1.61 (−3.02, 0.20) AU, *P* = 0.025)(Table 2). RMR, FFM and FM were not associated with any other elements of the CoEQ. These findings were similar in Model 1 (Supplementary Table 1).

### Food preference and reward (LFPQ)

In Model 2, RMR, FFM and FM were not associated with any outcomes within the LFPQ (Supplementary Table 2). These findings were similar in Model 1 (Supplementary Table 1).

### Taste and smell scores

In Model 2, FFM was positively associated with smell scores (0.07 (0.01, 0.12) AU, *P* = 0.026) but not related to taste (*P* = 0.313). FM was inversely associated with taste scores (−0.20 (−0.29, 0.10) AU, *P* < 0.001) whilst an inverse relationship with smell scores approached statistical significance (−0.07 (−0.14, 0.00) AU, *P* = 0.058). This pattern of results was similar in Model 1.

### Interaction analyses

Analyses exploring interactions in the association between RMR and key study outcomes are presented in Supplementary Table 3. The positive association between RMR and energy intake was stronger in younger individuals (*P* = 0.020), men (*P* = 0.004), those with lower BMI (*P* = 0.019), more physically active individuals (*P* = 0.005), and smokers (*P* = 0.001). The inverse association between RMR and fasting ghrelin concentrations was stronger in younger people (*P* < 0.001), men (*P* < 0.001), active individuals (*P* = 0.022), and smokers (*P* = 0.032). The inverse association between RMR and fasting PFC ratings was stronger in older people (*P* = 0.002), women (*P* = 0.022), those with higher BMI (*P* = 0.002), active individuals (*P* = 0.031), and non-smokers (*P* = 0.012).

Analyses exploring interactions in the association between FFM and key study outcomes are presented in Supplementary Table 4. The positive association of FFM with hunger AUC was stronger in older individuals (*P* = 0.026), those with higher BMIs (*P* = 0.041), more active people (*P* = 0.010) and non-smokers (*P* < 0.001). The positive association between FFM and PYY AUC was stronger in older individuals (*P* = 0.001), men (*P* = 0.002), people with lower BMIs (*P* = 0.012) and non-smokers (*P* = 0.004). The positive association between FFM and smell scores was stronger in people with lower BMIs (*P* = 0.011), while the positive association of FFM and craving control was stronger in older individuals (*P* = 0.007) and non-smokers (*P* = 0.003).

Analyses exploring interactions in the association between FM and key study outcomes are presented in Supplementary Table 5. The positive association of FM and circulating leptin was stronger in older people (*P* < 0.001), women (*P* < 0.001), those with lower BMI (*P* = 0.008), more active people (*P* < 0.001), and non-smokers (*P* < 0.001). The inverse association between FM and PYY AUC was stronger in older people (*P* = 0.003), men (*P* = 0.003), those with lower BMI (*P* = 0.003), less active people (*P* = 0.009), and non-smokers (*P* = 0.006). The inverse association between FM and hunger AUC was stronger in older people (*P* = 0.046), more active people (*P* = 0.014), and non-smokers (*P* = 0.002). The inverse association between FM and craving control was stronger in older people (*P* = 0.011), more active people (*P* = 0.011), and smokers (*P* = 0.036). The inverse association between FM and taste scores was stronger in those with higher BMIs (*P* = 0.048).

## Discussion

In this study, RMR was positively associated with laboratory-measured energy intake, whereas FFM and FM were not. These findings align with previous research in healthy, weight-stable individuals identifying RMR as a key physiological driver of daily intake, reflecting metabolic need [5, 34], but showing no association with FM [2, 5–7, 34, 35]. Contrary to earlier studies, we observed no relationship between FFM and energy intake [2, 5–7, 34, 35], which may be due to methodological factors such as the sensitivity of FFM measurement (via air displacement plethysmography), the constrained nature of lab-based intake assessments, or sample homogeneity. Notably, previous work suggests the association between FFM and energy intake is largely (up to 96%) or fully mediated by RMR [5, 35]. In the present cohort, however, only a small positive association was observed between FFM and RMR (*r* = 0.176; *P* = 0.048) which may help explain why FFM was not independently associated with energy intake. Collectively, these findings underscore the primacy of energy expenditure, rather than body composition, as a driver of daily energy intake.

It was recently suggested that associations of RMR with energy intake may vary depending on factors such as age, sex, and adiposity [3]; a notion we explored in greater detail. Interestingly, sex and BMI moderated the relationships between RMR and energy intake, such that the positive association between RMR and energy intake was stronger in men and individuals with lower BMI. The weaker associations in women may reflect undetermined biological differences in how metabolic demand relates to energy intake [36]; or a greater propensity for social desirability bias [37]. Equally, menstrual cycle phase was not controlled for, which could also potentially weaken the association in women—as daily energy intake may vary by ~275 kcal/d between luteal vs. follicular phases [38]. Like previous research [2, 6], the stronger RMR-energy intake coupling in leaner individuals may indicate greater sensitivity to metabolic energy requirements versus those with higher BMIs, where FM is disproportionately increased relative to FFM [2]. Equally, people with higher BMIs may be more sensitive to hedonic signals [6].

Other interesting findings were that the positive association between RMR and energy intake was stronger in more active individuals and in smokers. This may be related to better appetite sensitivity in active individuals [39], linked to higher energy flux [40]. In smokers, the stronger RMR–intake relationship could reflect the metabolic effects of nicotine, increasing energy expenditure [41] and driving energy intake, promoting closer coupling. Additionally, because smokers often report lower dietary restraint [42], their lab-measured intake may more accurately reflect their usual behaviour.

A key novelty of this study was testing whether RMR, FFM, and FM relate to homoeostatic and hedonic drivers of intake. Notably, we comprehensively examined associations with subjective appetite ratings and key appetite-related hormones –leptin, acylated ghrelin and PYY– in fasted and postprandial states. Overall, links with appetite ratings were limited, though FFM was positively, and FM was inversely associated with hunger AUC. FFM may reflect metabolic demand translating into eating behaviour, while FM’s inverse link may involve leptin, which increases with fat mass [43] and inhibits appetite [44]. RMR showed no such association, possibly due to greater measurement variability. The broader disconnect may reflect the complex, psychologically-mediated nature of appetite and individual differences in perception/reporting.

We also found that fasting leptin was positively associated with FM and inversely associated with FFM, while no associations were observed with RMR. The positive link with FM is expected, as leptin is produced by adipose tissue and increases with adiposity [43], while the inverse relationship with FFM may reflect lower FM typically observed in individuals with higher FFM. Interaction analysis showed that several variables moderated the opposing associations of FFM and FM with leptin, including age, sex, BMI and smoking status. Accordingly, stronger associations in older individuals may be linked to age-related changes in adipose tissue function and increased leptin resistance, leading to disproportionately elevated leptin concentrations at higher FM [45]. In women, stronger associations could arise from a higher proportion of subcutaneous adipose tissue and greater leptin production per unit of FM [46]. The stronger associations in non-smokers and individuals with lower BMI likely reflect a tighter coupling between FM and leptin in the absence of nicotine-related alteration to leptin signalling [47] and greater leptin sensitivity at lower adiposity [48], respectively.

In this study, the postprandial PYY response was positively associated with FFM and negatively associated with FM, while fasting PYY positively associated with FM. The inverse association of PYY AUC with FM supports previous findings of blunted PYY meal responses in individuals with obesity [49], suggesting impaired satiety signalling with increasing adiposity. The positive association of PYY AUC with FFM aligns with findings from a prior study in bariatric surgery patients [50, 51], possibly reflecting PYY expression in skeletal muscle [52, 53]^.^ The positive association between fasting PYY and FM is less clear, as PYY is typically lower in people with higher adiposity [49]. However, two previous studies noted this relationship, with proposed explanations including a protective (compensatory) response to increased adiposity and/or possible PYY resistance [54, 55]. Further research is needed to clarify these hypotheses.

The opposing relationships of FFM and FM with PYY AUC were moderated by age, sex, BMI and smoking status, with stronger relationships in the older, male, lower BMI and non-smoking sub-groups. These patterns suggest that demographic, anthropometric, and lifestyle factors influence meal-related PYY responses, potentially through differences in gut peptide secretion, receptor sensitivity, or metabolic function [11]. The stronger FFM-PYY association in older adults could represent a compensatory response to age-related changes in appetite [7], or its link with skeletal muscle, which typically declines in older age [7, 53]. The weaker associations in smokers are consistent with the known suppressive effects of nicotine on gut hormone responses [35, 48].

For ghrelin, an inverse association was observed between RMR and fasting ghrelin, with stronger relationships in individuals who were younger, men, more active and smokers. Lower fasting ghrelin in individuals with higher RMR is consistent with previous research and, given the weight-stable status of participants, suggests ghrelin may be shaped by habitual energy flux/turnover rather than metabolic demand alone [3, 4, 12]. The stronger associations in more active individuals [40] and smokers [35, 48] further support the influence of lifestyle factors on fasting ghrelin. Given the lack of postprandial associations and the centrality of postprandial dynamics in appetite regulation, the practical significance of these findings warrants further investigation.

Beyond these homoeostatic drivers, we examined hedonic aspects of eating behaviour using the CoEQ, which assesses craving intensity, control and emotional eating, and the LFPQ, which captures both explicit and implicit food preferences across fat and sweet domains [29]. We found that FFM (positively) and FM (negatively) were associated with craving control, while no other relationships were apparent for the CoEQ and LFPQ components. While it is possible that biological or psychological factors could link FM to craving control, the reverse relationship may also be true: people with weaker craving control may simply gain more FM due to poorer self-regulation [56]. Further, the opposite (positive) association between FFM and craving control may reflect lower FM in people with greater FFM.

Interestingly, the inverse relationship between FM and craving control was stronger in older adults, more active individuals, and smokers, suggesting that in these subgroups, elevated FM may more clearly indicate long-term appetite dysregulation. In older adults, age-related declines in executive function may amplify the impact of FM on hedonic eating [57]. Among active individuals, who generally exhibit better appetite regulation and higher dietary restraint, higher FM may reflect a mismatch between expected regulation and actual behaviour, accentuating poor craving control. In smokers, where nicotine exerts an inhibitory influence on appetite, and dietary restraint is typically lower [27], high FM may more directly signal impaired self-regulation and heightened susceptibility to cravings.

To complement our hedonic analysis, we assessed taste and smell sensitivity, finding positive associations of FFM with smell sensitivity, while FM was inversely related to smell (tendency) and taste sensitivity. No associations were apparent with RMR, suggesting that gustatory and olfactory function are more closely linked to body composition than energy demand. While previous studies have linked higher FM and/or obesity to poorer taste [58] and smell sensitivity [52], we extend this by showing that associations between FM and taste perception are modified by BMI, with the relationship being stronger in people with higher BMI. This may be due to alterations in adipokines with increasing adiposity, which inhibit these senses [53, 59]. Mechanisms underpinning the positive FFM-smell relationship remain unclear but may reflect lower FM in those with greater lean mass rather than a distinct biological mechanism.

A key strength of this study is its comprehensive phenotyping, combining objective measures of body composition, RMR, and diverse appetite-related outcomes in weight-stable individuals. This multidimensional approach enhances understanding of appetite regulation beyond intake alone. However, the cross-sectional design limits causal inference, and the young, lean, active sample reduces generalisability. Findings may not apply to weight change contexts, and lack of menstrual cycle control could have introduced variability among females. Finally, while validated behavioural tools were used to assess hedonic appetite, future studies incorporating neuroimaging approaches (e.g. functional MRI) may provide additional insight into central reward processes.

In conclusion, we found that RMR is positively associated with lab-measured energy intake, with the relationship moderated by sex, BMI, physical activity, and smoking. FFM and FM were also linked to appetite-related hormones and hedonic traits, particularly craving control and taste perception. Notably, the inverse association of FM with craving control was stronger in the older, more active, and smoking sub-groups, indicating higher FM in these individuals may reflect greater appetite dysregulation. These findings highlight the complex interplay between body composition, energy demand, and homoeostatic and hedonic drivers of appetite.

## Supplementary information

Supplementary materials

## Data Availability

The data that support the findings of this study are available from the corresponding author upon reasonable request.
