# Supplementary material for: Associations of body composition and resting metabolic rate with homeostatic and hedonic components of appetite
Source: Int J Obes (Lond). 2026 Jun 2;50(9):1945–52. doi: 10.1038/s41366-026-02115-1 (PMC13649550; doi:10.1038/s41366-026-02115-1)
Supplement: Supplementary file 1 [file 41366_2026_2115_MOESM1_ESM.docx]

**Supplementary Table 1. Associations of resting metabolic rate, fat−free mass and fat mass with energy intake, appetite−related hormones, perceived ratings of appetite, taste and smell, and control of eating, in model 1 (adjusted for age, sex, and ethnicity).**

|  | **Resting metabolic rate (kcal·d^−1^)** | | **Fat−free mass (kg)** | | **Fat mass (kg)** | |
| --- | --- | --- | --- | --- | --- | --- |
|  | **β (95% CI)** | ***P−*value** | **β (95% CI)** | ***P−*value** | **β (95% CI)** | ***P−*value** |
| **Energy intake** | | | | | | |
| Lab energy intake (kcal·d^−1^) | 6.4 (3.1, 9.7) | **< 0.001** | 3.9 (−6.8, 14.4) | 0.484 | 0.2 (−11.2, 11.5) | 0.975 |
| Field energy intake (kcal·d−1) | 7.9 (3.8, 12.2) | **< 0.001** | 4.3 (−9.3, 18.0) | 0.534 | −1.3 (−15.9, 13.3) | 0.865 |
| **Appetite−related hormones** | | | | | | |
| Fasting ghrelin (pg·mL^−1^)^*^ | 0.00 (−0.00, 1.00) | 0.152 | 0.01 (−0.02, 0.10) | 0.488 | 0.00 (−0.02, 0.02) | 0.984 |
| Ghrelin AUC (2 h, pg·mL^−1^) | −2.80 (−31.8, 26.20) | 0.850 | −12.70 (−101.69, 76.29) | 0.780 | 2.84 (−92.47, 98.14) | 0.953 |
| Fasting PYY (pg·mL^−1^) | −0.10 (−0.37, 0.27) | 0.743 | −0.33 (−1.30, 0.65) | 0.514 | 0.55 (−0.50, 1.59) | 0.306 |
| PYY AUC (2 h, pg·mL^−1^) | −11.17 (−37.18, 14.84) | 0.400 | 116.87 (39.24, 194.50) | **0.003** | 2.84 (−92.47, 98.14) | 0.953 |
| Fasting leptin (pg·mL^−1^)^*^ | 0.00 (0.00, 0.00) | 0.186 | −0.06 (−0.08, −0.05) | **< 0.001** | 0.07 (0.05, 0.08) | **< 0.001** |
| **Perceived ratings of appetite** | | | | | | |
| Fasting fullness (mm) | 0.04 (−0.10, 0.17) | 0.510 | −0.28 (−0.67, 0.12) | 0.174 | 0.33 (−0.09, 0.76) | 0.124 |
| Fullness AUC (4 h, mm) | −0.16 (−24.93, 24.60) | 0.990 | −65.74 (−141.37, 9.88) | 0.088 | 66.40 (−14.73, 147.54) | 0.109 |
| Fasting hunger (mm) | −0.10 (−0.21, 0.10) | 0.506 | 0.00 (−0.147, 0.48) | 0.987 | 0.01 (−0.50, 0.51) | 0.973 |
| Hunger AUC (4 h, mm) | 10.55 (−15.97, 37.08) | 0.436 | 89.65 (9.60, 169.69) | 0.028 | −88.21 (−174.06, −2.37) | **0.044** |
| Fasting PFC (mm) | −0.11 (−0.22, −0.01) | **0.030** | 0.14 (−0.18, 0.46) | 0.387 | −0.15 (−0.49, 0.91) | 0.395 |
| PFC AUC (4 h, mm) | 13.80 (−9.21, 36.81) | 0.240 | 63.42 (−5.72, 132.57) | 0.072 | −57.69 (−131.88, 16.50) | 0.127 |
| Fasting satisfaction (mm) | 0.10 (−0.10, 0.20) | 0.401 | 0.19 (−0.24, 0.63) | 0.388 | −0.21 (−0.67, 0.26) | 0.385 |
| Satisfaction AUC (4 h, mm) | 17.72 (−6.10, 41,54) | 0.145 | −27.14 (−100.76, 46.49) | 0.470 | 27.80 (−50.95, 106.55) | 0.489 |
| **CoEQ** |  |  |  |  |  |  |
| Craving control (AU) | −0.03 (−0.07, 0.01) | 0.203 | 1.08 (0.58, 3.01) | **0.004** | −1.89 (−3.19, −0.59) | **0.004** |
| Craving for Sweet (AU) | 0.00 (−0.05, 0.05) | 0.998 | 0.73 (−0.81, 2.27) | 0.352 | −0.66 (−2.30, 0.99) | 0.432 |
| Craving for Savoury (AU) | 0.03 (−0.02, 0.08) | 0.208 | −0.87 (−2.31, 0.58) | 0.241 | 1.08 (−0.46, 2.62) | 0.170 |
| Positive Mood (AU) | 0.01 (−0.03, 0.04) | 0.806 | 1.27 (0.08, 2.45) | 0.036 | −1.17 (−2.44, 0.11) | 0.072 |
| **Food preference and reward** | | | | | | |
| Fasting fat explicit liking  (mm) | −0.06 (−0.14, −0.02) | 0.140 | −0.03 (−0.27, 0.21) | 0.814 | 0.02 (−0.24, 0.28) | 0.861 |
| Fasting fat explicit wanting  (mm) | −0.04 (−0.12, 0.04) | 0.317 | 0.02 (−0.23, 0.26) | 0.904 | −0.04 (−0.30, 0.23) | 0.788 |
| Fasting fat implicit wanting  (AU) | −0.07 (−0.23, 0.09) | 0.396 | −0.15 (−0.65, 0.36) | 0.566 | 0.14 (−0.40, 0.68) | 0.615 |
| Fasting fat relative preference  (AU) | −0.03 (−0.09, 0.03) | 0.342 | 0.05 (−0.23, 0.14) | 0.640 | 0.03 (−0.17, 0.23) | 0.740 |
| Fasting taste explicit liking  (mm) | 0.01 (−0.09, 0.12) | 0.794 | 0.16 (−0.17, 0.49) | 0.347 | −0.18 (−0.54, −0.17) | 0.311 |
| Fasting taste explicit wanting  (mm) | 0.01 (−0.09, 0.12) | 0.828 | 0.10 (−0.23, 0.42) | 0.556 | −0.11 (−0.46, 0.24) | 0.532 |
| Fasting taste implicit wanting  (AU) | −0.06 (−0.28, 0.16) | 0.601 | 0.34 (−0.35, 1.03) | 0.330 | −0.46 (−1.20, 0.27) | 0.217 |
| Fasting taste relative preference  (AU) | 0.01 (−0.07, 0.009) | 0.861 | 0.08 (−0.17, 0.33) | 0.525 | −0.10 (−0.37, 0.17) | 0.454 |
| **Taste and smell** |  |  |  |  |  |  |
| Taste score (AU) | −0.03 (−0.06, 0.00) | 0.068 | 0.10 (−0.05, 0.25) | 0.199 | −0.14 (−0.32, −0.05) | 0.154 |
| Smell score (AU) | 0.00 (−0.01, 0.01) | 0.931 | 0.07 (0.02, 0.13) | **0.010** | −0.07 (−0.14, −0.01) | **0.021** |

Data were analysed using generalised linear models and are presented as beta−coefficients (β) with 95% confidence intervals (CI). Models for most outcomes used a normal distribution and identity link function, however, based on model fit, skewed outcomes used a gamma distribution with either an identity link function^a^ or log link function^b^. Model 1 was adjusted for age, sex, ethnicity, and fasting values (for AUC variables). *P* < 0.05 indicates a statistically significant association. AU, arbitrary units; AUC, area under the curve; PFC, prospective food consumption; PYY, peptide YY, ^*^ β−coefficients for fasting leptin and fasting ghrelin are based on log−transformed value

**Supplementary Table 2. Associations of resting metabolic rate, fat−free mass and fat mass with food preference and reward, in model 2 (adjusted for age, sex, and ethnicity, and lifestyle).**

|  | **Resting metabolic rate (kcal·d^−1^)** | | **Fat−free mass (kg)** | | **Fat mass (kg)** | |
| --- | --- | --- | --- | --- | --- | --- |
|  | **β (95% CI)** | ***P−*value** | **β (95% CI)** | ***P−*value** | **β (95% CI)** | ***P−*value** |
| **Food preference and reward** | | | | | | |
| Fasting fat explicit liking  (mm) | –0.06 (–0.14, 0.02) | 0.128 | 0.03 (–0.23, 0.29) | 0.808 | –0.04 (–0.32, 0.24) | 0.762 |
| Fasting fat explicit wanting  (mm) | –0.05 (–0.13, 0.04) | 0.260 | 0.05 (–0.21, 0.32) | 0.696 | –0.08 (–0.37, 0.21) | 0.582 |
| Fasting fat implicit wanting  (AU) | –0.08 (–0.25, 0.09) | 0.347 | –0.05 (–0.59, 0.49) | 0.856 | 0.02 (–0.56, 0.61) | 0.935 |
| Fasting fat relative preference  (AU) | –0.03 (–0.10, 0.03) | 0.309 | 0.02 (–0.22, 0.19) | 0.880 | –0.003 (–0.22, 0.21) | 0.975 |
| Fasting taste explicit liking  (mm) | 0.03 (–0.08, 0.15) | 0.543 | 0.11 (–0.24, 0.47) | 0.533 | –0.14 (–0.52, 0.24) | 0.475 |
| Fasting taste explicit wanting  (mm) | 0.02 (–0.09, 0.13) | 0.665 | 0.05 (–0.30, 0.40) | 0.780 | –0.06 (–0.44, 0.31) | 0.739 |
| Fasting taste implicit wanting  (AU) | –0.02 (–0.25, 0.21) | 0.883 | 0.34 (–0.39, 1.07) | 0.365 | –0.49 (–1.27, 0.30) | 0.227 |
| Fasting taste relative preference  (AU) | 0.02 (–0.07, 0.10) | 0.661 | 0.07 (–0.20, 0.33) | 0.632 | –0.09 (–0.38, 0.20) | 0.538 |

Data were analysed using generalised linear models and are presented as beta−coefficients (β) with 95% confidence intervals (CI). Models for most outcomes used a normal distribution and identity link function. The finally adjusted models (model 2) were adjusted for age, sex, ethnicity, *V̇*O_2_ peak, moderate−vigorous physical activity, accelerometer wear time, smoking status and fasting values (for AUC variables). *P* < 0.05 indicates a statistically significant association. AU, Arbitrary Unit

**Supplementary Table 3. Interactions of demographic, anthropometric, and lifestyle factors in the association between resting metabolic rate and key study outcomes.**

|  |  | **Category 1 β (95% CI)** | **Category 2 β (95% CI)** |
| --- | --- | --- | --- |
|  | ***P−*value for**  **interaction** | |  |
| **Interactions with Age** |  | **< 25.5 years** | **≥ 25.5 years** |
| Fasting PFC (mm)  Fasting ghrelin (pg·mL^−1^)  Lab energy intake (kcal·d−1) | **0.002**  **<0.001**  **0.020** | –0.12 (–0.26, 0.02)  –0.10 (–0.10, 0.00)  5.88 (2.01, 9.75) | –0.15 (–0.30, 0.01)  0.00 (–0.10, 0.00)  5.50 (0.33, 10.66) |
| Field energy intake (kcal·d−1) | **0.039** | 5.47 (−0.60, 11.53) | 6.29 (1.07, 11.52) |
| **Interactions with Sex** |  | **Male** | **Female** |
| Fasting PFC (mm)  Fasting ghrelin (pg·mL^−1^)  Lab energy intake (kcal·d−1) | **0.022**  **<0.001**  **0.004** | 0.18 (−0.31, 0.05)  −0.01 (−0.01, 0.00)  5.74 (1.46, 10.03) | −0.01 (−0.23, 0.21)  0.00 (−0.01, 0.01)  5.47 (−0.68, 11.62) |
| Field energy intake (kcal·d−1) | **0.023** | 5.39 (−0.46, 11.24) | 7.05 (0.64, 13.45) |
| **Interactions with BMI** | | **< 23.55 kg·m^−2^** | **≥ 23.55 kg·m^−2^** |
| Lab energy intake (kcal·d^−1^) | **0.019** | 7.85 (3.29, 12.42) | 2.76 (−2.63, 8.15) |
| Field energy intake (kcal·d−1) | **0.008** | 4.30 (−1.40, 10.00) | 7.09 (0.41, 13.78) |
| Fasting PFC (mm) | **0.002** | −0.08 (−0.26, 0.09) | −0.26 (−0.38, −0.14) |
| **Interactions with MVPA** |  | **< 108.83 mins·d^−1^** | **≥ 108.83 mins·d^−1^** |
| Lab energy intake (kcal·d^−1^) | **0.005** | 0.528 (–0.49, 1.10) | 6.18 (2.36, 10.00) |
| Fasting PFC (mm)  Fasting ghrelin (pg·mL^−1^) | **0.031**  **0.022** | 0.00 (0.00, 0.01)  0.00 (–0.01, 0.00) | 0.00 (–0.01, 0.01)  –0.10 (–0.10, –0.01) |
| **Interactions with CRF** |  | **< 44.51 ml·kg^−1^·min^−1^** | **≥ 44.51 ml·kg^−1^·min^−1^** |
| Lab energy intake (kcal·d^−1^) | **0.031** | 8.26 (3.90, 12.61) | 3.80 (–1.20, 8.80) |
| Field energy intake (kcal·d−1) | **0.031** | 7.84 (1.71, 13.96) | 2.38 (−3.55, 8.32) |
| Fasting PFC (mm) | **0.005** | −0.14 (−0.30, 0.01) | –0.12 (–0.27, 0.02) |
| **Interactions with Smoking** | | **Smokers** | **Non−smokers** |
| Lab energy intake (kcal·d^−1^) | **0.001** | 11.86 (7.72, 16.00) | 3.43 (−1.08, 0.79) |
| Field energy intake (kcal·d−1) | **0.004** | 13.12 (4.38, 21.87) | 1.66 (−3.15, 6.47) |
| Fasting PFC (mm) | **0.012** | 0.04 (−0.12, 0.20) | −0.21 (−0.35, −0.07) |
| Fasting ghrelin (pg·mL^−1^) | **0.032** | −0.01 (−0.01, 0.00) | 0.00 (−0.01, 0.00) |

Data are presented as *P*−values for the interaction term and as β−coefficients (95% confidence intervals) for categorical variables and continuous variables stratified using a median split. Models were adjusted for age, sex, ethnicity, MVPA, device wear time, CRF and smoking status. AU, arbitrary unit; BMI, body mass index; CI, confidence interval; CRF, cardiorespiratory fitness, ^*^ β−coefficients for fasting leptin is based on log−transformed value

**Supplementary Table 4. Interactions of demographic, anthropometric, and lifestyle factors in the association between fat−free mass and key study outcomes.**

|  |  | **Category 1 β (95% CI)** | **Category 2 β (95% CI)** |
| --- | --- | --- | --- |
|  | ***P−*value for**  **interaction** | |  |
| **Interactions with Age** |  | **< 25.5 years** | **≥ 25.5 years** |
| PYY AUC (pg·mL^−1^) | **0.001** | 97.77 (−29.98, 225.51) | 192.24 (76.83, 307.64) |
| Fasting leptin (pg·mL^−1^) | **<0.001** | −462.23 (−678.77, −245.69) | −550.07 (−828.13, −272.01) |
| Craving Control | **0.007** | 0.37 (−1.63, 2.37) | 2.32 (0.68, 3.96) |
| Hunger AUC (4 h, mm) | **0.026** | 89.55 (−38.67, 217.76) | 93.90 (−28.37, 216.17) |
| **Interactions with Sex** |  | **Male** | **Female** |
| PYY AUC (pg·mL^−1^) | **0.002** | 177.23 (59.29, 295.18) | 127.88 (16.27, 239.50) |
| Fasting leptin (pg·mL^−1^) | **<0.001** | −253.62 (−331.83, −175.40) | −799.87 (−1190.23, −409.51) |
| **Interactions with BMI** |  | **< 23.55 kg·m^−2^** | **≥ 23.55 kg·m^−2^** |
| Fasting leptin (pg·mL^−1^) | **0.003** | −486.16 (−770.19, −202.13) | –163.55 (–461.34, 134.25) |
| PYY AUC (pg·mL^−1^) | **0.012** | 279.45 (96.04, 462.87) | 106.56 (–26.92, 240.04) |
| Smell score (AU) | **0.011** | 0.11 (–0.01, 0.23) | 0.10 (0.02, 0.18) |
| Hunger AUC (4 h, mm) | **0.041** | 97.52 (–70.99, 266.03) | 132.38 (–27.91, 292.67) |
| **Interactions with MVPA** |  | **< 108.83 mins·d^−1^** | **≥ 108.83 mins·d^−1^** |
| Hunger AUC (4 h, mm) | **0.010** | 83.51 (−39.79, 206.81) | 125.08 (−6.24, 256.39) |
| **Interactions with CRF** |  | **< 44.51 ml·kg^−1^·min^−1^** | **≥ 44.51 ml·kg^−1^·min^−1^** |
| PYY AUC (pg·mL^−1^) | **0.001** | 116.86 (7.99, 225.74) | 169.94 (34.83, 305.04) |
| Fasting leptin (pg·mL^−1^) | **<0.001** | −665.40 (−940.20, −390.59) | −377.39 (−565.78, −189.00) |
| Craving Control | **0.009** | 0.28 (−1.53, 2.09) | 3.54 (1.55, 5.52) |
| Hunger AUC (4 h, mm) | **0.025** | 38.50 (−93.20, 170.19) | 129.66 (2.39, 256.93) |
| **Interactions with Smoking Status** | | **Smokers** | **Non−smokers** |
| PYY AUC (pg·mL^−1^) | **0.004** | 79.60 (−34.40, 193.60) | 177.43 (66.04, 288.82) |
| Fasting leptin (pg·mL−1) | **<0.001** | −510.67 (−782.23, −239.11) | −560.86 (−775.74, −345.97) |
| Craving Control | **0.003** | 1.15 (−0.84, 3.15) | 2.07 (0.48, 3.66) |
| Hunger AUC (4 h, mm) | **<0.001** | –48.68 (–213.69, 116.32) | 196.64 (90.58, 302.70) |
|  |  |  |  |

Data are presented as *P*−values for the interaction term and as β−coefficients (95% confidence intervals) for categorical variables and continuous variables stratified using a median split. Models were adjusted for age, sex, ethnicity, MVPA, device wear time, CRF and smoking status. AU, arbitrary unit; BMI, body mass index; CI, confidence interval; CRF, cardiorespiratory fitness; MVPA, moderate−vigorous physical activity, ^*^ β−coefficients for fasting leptin is based on log−transformed values.

**Supplementary Table 5. Interactions of demographic, anthropometric, and lifestyle factors in the association between fat mass and key study outcomes.**

|  |  | **Category 1 β (95% CI)** | **Category 2 β (95% CI)** |
| --- | --- | --- | --- |
|  | ***P−*value for**  **interaction** | |  |
| **Interaction with Age** | | **< 25.5 years** | **≥ 25.5 years** |
| PYY AUC (pg·mL^−1^) | **0.003** | −108.52 (–249.18, 32.15) | −202.28 (−329.16, −75.40) |
| Fasting leptin (pg·mL^−1^) | **<0.001** | 458.57 (216.57, 700.58) | 725.18 (437.67, 1012.68) |
| Hunger AUC (4 h, mm) | **0.046** | –72.06 (–213.84, 69.72) | −101.52 (−235.80, 32.76) |
| Craving Control | **0.011** | –0.36 (–2.55, 1.83) | −2.53 (−4.34, −0.73) |
| **Interactions with Sex** | | **Male** | **Female** |
| PYY AUC (pg·mL^−1^) | **0.003** | −192.60 (−318.01, −67.18) | −132.59 (–261.88, –3.29) |
| Fasting leptin (pg·mL^−1^) | **<0.001** | 272.53 (189.73, 355.33) | 1054.75 (646.33, 1463.18) |
| **Interactions with BMI** | | **< 23.55 kg·m^−2^** | **≥ 23.55 kg·m^−2^** |
| PYY AUC (pg·mL^−1^) | **0.003** | −278.09 (−512.41, −43.77) | −128.55 (−256.92, −0.18) |
| Fasting leptin (pg·mL^−1^) | **0.008** | 640.83 (191.25, 1090.40) | 222.75 (−135.33, 580.83) |
| Taste Score (AU) | **0.048** | −0.08 (−0.36, 0.20) | −0.17 (−0.33, −0.01) |
| **Interactions with MVPA** | | **< 108.83 mins·d^−1^** | **≥ 108.83 mins·d^−1^** |
| PYY AUC (pg·mL^−1^) | **0.009** | −198.80 (–331.56, –66.04) | −73.39 (–192.71, 45.93) |
| Fasting leptin (pg·mL^−1^) | **<0.001** | 573.97 (321.30, 826.65) | 616.14 (367.87, 864.40) |
| Hunger AUC (4 h, mm) | **0.014** | −96.20 (–229.93, 37.53) | −102.22 (–242.27, 37.83) |
| Craving Control | **0.011** | –0.46 (–2.01, 1.10) | −3.20 (–5.48, –0.92) |
| **Interactions with CRF** | | **< 44.51 ml·kg^−1^·min^−1^** | **≥ 44.51 ml·kg^−1^·min^−1^** |
| PYY AUC (pg·mL^−1^) | **0.002** | –124.81 (–238.73, –10.89) | –193.50 (–351.17, –35.83) |
| Fasting leptin (pg·mL^−1^) | **<0.001** | 748.21 (470.07, 1026.35) | 478.81 (264.52, 693.09) |
| Hunger AUC (4 h, mm) | **0.048** | –49.50 (–187.27, 88.28) | –107.74 (–258.04, 42.56) |
| **Interactions with Smoking Status** | | **Smokers** | **Non−smokers** |
| PYY AUC (pg·mL^−1^) | **0.006** | −47.96 (–172.91, 76.99) | −190.16 (–307.15, –73.16) |
| Fasting leptin (pg·mL^−1^) | **<0.001** | 543.79 (252.38, 835.19) | 645.72 (427.36, 864.08) |
| Hunger AUC (4 h, mm) | **0.002** | 101.14 (–75.44, 277.73) | –193.98 (–305.67, –82.29) |
| Craving Control | **0.036** | –2.01 (–4.07, 0.05) | –1.67 (–3.35, 0.02) |

Data are presented as *P*−values for the interaction term and as β−coefficients (95% confidence intervals) for categorical variables and continuous variables stratified using a median split. Models were adjusted for age, sex, ethnicity, MVPA, device wear time, CRF and smoking status. AU, arbitrary unit; BMI, body mass index; CI, confidence interval; CRF, cardiorespiratory fitness, ^*^ β−coefficients for fasting leptin is based on log−transformed value
